# Optimization of multiple enzymes production by fermentation using lipid-producing *Bacillus* sp.

**DOI:** 10.3389/fmicb.2022.1049692

**Published:** 2022-11-01

**Authors:** Sarita Shrestha, Chonlong Chio, Janak Raj Khatiwada, Aristide Laurel Mokale Kognou, Wensheng Qin

**Affiliations:** Department of Biology, Lakehead University, Thunder Bay, ON, Canada

**Keywords:** *Bacillus* sp., multi-enzymes, agro-wastes, optimization, lipid content

## Abstract

The present study identified the pectinase-producing bacterium isolated from the contaminated broth as *Bacillus* sp. on 16S rDNA sequence analysis. The bacterium illustrated water-like droplets on the colony grown on the Sabouraud dextrose agar plate. It also exhibited multi-enzymes activities, such as pectinase, polygalacturonase, xylanase, and cellulase by using various agro-wastes as low-cost substrates. The orange peel was observed to be the best substrate among the agro-wastes used for maximum multi-enzymes (pectinase, polygalacturonase, xylanase, and cellulase). However, the bacterium demonstrated its capability to produce different enzymes according to the different substrates/agro-wastes used. The Plackett–Burman design was used to determine the essential influencing factors, while the Box Behnken design response surface methodology was for optimizing cultural conditions. At their optimal conditions (40°C incubation temperature, 24 h of incubation period, 1% w/v orange peel, and 2% v/v inoculum volume), the bacterium exhibited the maximum pectinase (9.49 ± 1.25 U/ml) and xylanase (16.27 ± 0.52 U/ml) activities. Furthermore, the study explored the ability of the bacterium to produce bacterial lipids and observed about 25% bacterial lipid content on a dry weight basis. Therefore, the bacterium is a good candidate for producing important multi-enzymes and subsequent agro-waste degradation controlling the environment, and facilitating waste management. Also, the bacterium can be a potential feedstock in producing renewable biofuel.

## Introduction

Agro-wastes are low-cost, renewable, and sustainable resources for industrially important enzyme production (Bharathiraja et al., 2017; Ravindran et al., 2018; Shrestha et al., 2020). Agricultural waste is usually produced during processing, pre-harvesting, post-harvesting, marketing procedures, and household activities (Kodagoda and Marapana, 2017; Shrestha et al., 2021a). The loss of agricultural products during these processes may decrease the products and increase production costs. Additionally, the agro-wastes pollute the environment and create disposal problems (Sadh et al., 2018; Shrestha et al., 2021a). Population growth and waste generation are directly proportional and it is expected the waste generation per year will increase by 83% by 2050.^1^ Dumping or landfill is the common method of waste disposal. However, it is not safe. The air near or surrounding the area of the dumping site may have air pollution due to the gasses produced and the suspended particles in the air (Siddiqua et al., 2022). The leachate from landfill may contain toxic byproducts and may contaminate water bodies or soil which finally may affect living things including human and aquatic life (Parvin and Tareq, 2021; Siddiqua et al., 2022). At the same time, there is a continuous rise in demand for enzymes, and the pure carbon and nitrogen source used in enzyme production is expensive (Ravindran et al., 2018; Shrestha et al., 2022). Therefore, these agro-wastes can be used as low-cost substrates to produce polysaccharides hydrolyzing enzymes as they contain different polysaccharides such as cellulose, xylose, and pectin in different compositions (Sadh et al., 2018; Mellinas et al., 2020; Shrestha et al., 2020, 2022).

Besides, the multi-enzymes biocatalyst technology and production of enzyme cocktails from a single organism are gaining interest (Amadi et al., 2022). The most common and commercially important enzymes are cellulase, pectinase, and xylanase, used for broad biotechnological applications. Pectinases and xylanases have been used in wastewater treatment, brewing technology, animal feed preparation, textile, pulp and paper industries, and food processing industries (Thite et al., 2020; Nawawi et al., 2022). Cellulolytic enzymes and other enzymes apply to cell wall disruption, juice extraction, and lipid extraction (Guo et al., 2017). In addition, the multi-enzymes help protects the environment by degrading various plant and agro-wastes (Ravindran et al., 2018; Thite et al., 2020; Amadi et al., 2022; Shrestha et al., 2022). Furthermore, enzymes production depends on the microorganisms exploited, and there are only a few studies for multi-enzymes productions from a single bacterium (Laothanachareon et al., 2022; Ozzeybek and Cekmecelioglu, 2022; Shrestha et al., 2022). Producing multi-enzymes by a single bacterium using different agro-wastes is cost-effective and time-efficient because using a single pure carbohydrate as substrate can only induce a single specific enzyme which is expensive and time-consuming (Wang et al., 2019; Thite et al., 2020; Nawawi et al., 2022; Shrestha et al., 2022).

Furthermore, the uninterrupted increase in the world’s population is diminishing fossil fuel reserves, so there is a need to explore alternative energy sources (Yusuf, 2019). In this aspect, some oleaginous microorganisms are natural oil producers. They accumulate about 20% w/v of lipid on a dry weight basis and are the most promising feedstock for lipid and oleochemical production (Patel et al., 2020). Therefore, this study focuses on the isolation and identification of the bacterium that increased the pectinase activity significantly. Also, the current study exploits the bacterium for multi-enzymes production by utilizing different agro-wastes and optimization of the fermentation conditions. Furthermore, the bacterium was exploited to know its capability of producing lipids due to the unique colony of the bacterium with water-like droplets on Sabouraud dextrose agar plate.

## Materials and methods

### Isolation and identification of bacteria

The bacterium was isolated from the contaminated broth. A loopful of the contaminated broth was streaked on Sabouraud dextrose agar (SDA) and nutrient agar (NA) plates and incubated at room temperature and 35°C, respectively, for 2–3 days. After the growth of colonies, a single colony was sub-cultured many times on SDA and NA plates to get the pure isolated colonies. Once the pure isolated colonies were observed on the agar plate, genomic DNA was extracted following the SDS-CTAB/NaCl method.

The 16S rDNA genes of the isolate were amplified by Taq DNA polymerase with universal primer sets: 16S rDNA forward primer 5′-AGAGTTTGATCCTGGCTCAG-3′ and reverse primer 5′-GGTTACCTTGTTACGACTT-3. The amplification systems for the 16S PCR reaction mixture contained 2 × *Taq* PCR master mix of 12 μl (10 × Taq DNA polymerase buffer, 10 mM dNTPs, 25 mM of MgCl_2_, 1 U of *Taq* DNA polymerase), 3 μl of 10 μM forward and reverse primers, 2 μl of the genomic DNA template, and 4 μl of distilled water, making a total volume of 24 μl. Furthermore, the PCR reaction conditions used were denaturation at 94°C for 5 min and the cycle starting at 94°C for 30 s, followed by annealing at 54°C for 30 s, extending at 72°C for 1.5 min for 33 cycles, and finally extending at 72°C for 10 min. Then the PCR products were determined by 1% (w/v) agarose gel electrophoresis. The 16S rDNA target fragments from the gel were cut, DNA was extracted using a gel extraction minipreps kit (Bio Basic), and the extracted DNA was sent for DNA sequencing.

#### Gene sequencing and phylogenetic analysis

The 16S rDNA sequences of the isolate provided by the sequencing company were compared with the known sequences found in the National Center for Biotechnology Information (NCBI) database using the basic local alignment search tool (BLASTn). The isolate was identified based on the percentage similarity with the known species sequences in the database. All the sequences were collected and parallelized using the Clustalw module in BioEdit v. 7.0.9.0 (Hall, 1999) with default settings. Phylogenetic analysis was performed using a Neighbor-Joining (NJ) tree with 1,000 bootstraps using MEGA 7 (Kumar et al., 2016).

#### Screening tests

Different screening tests for pectinase, cellulase, xylanase, and amylase were performed by culturing the bacterium on agar plates containing pectin, cellulose, xylan, and starch, respectively. After the growth of the bacterium, potassium iodide solution for pectinase, Congo red for cellulase and xylanase, and iodine solution for amylase screening test were flooded. The clear halo zone around the colonies indicated the presence of respective enzymes (Meddeb-Mouelhi et al., 2014; Takcı and Turkmen, 2016; Al Mousa et al., 2022).

#### Pectinase activity

The speck of pure isolated colony, which showed the clear pectinolytic zone on screening pectin agar plate, was subjected to prepare seed culture. Then, 1% v/v seed culture was inoculated into a flask with 50 ml of yeast extract pectin media (YEP) composed of 0.3% yeast extract, 1% pectin, 0.2% KH_2_PO_4,_ and 0.2% K_2_HPO_4_ in distilled water. The inoculated flasks were incubated at 35°C for 4 days at 200 rpm. Samples from inoculated flasks were collected at regular intervals of 24 h, and enzyme activities were assayed and compared. Pectinase activity was calculated by measuring the reducing sugar content released from the substrate following the 3,5-dinitrosalicylic acid (DNS) method (Miller, 1959), as mentioned in a previous study (Shrestha et al., 2021b). In brief, 10 μl of crude enzyme extract was added to 20 μl of 1% citrus pectin solution as a substrate solution in the wells of a microplate, incubated in a 50°C water bath for 10 min, cooled, and 60 μl of DNS reagent was added. Then the microplate was covered and heated in boiling water for 5 min, followed by cooling down to room temperature. To the mixture, 200 μl of distilled water was added, and the absorbance was recorded at 540 nm to calculate the amount of reducing sugar released. Each enzymatic activity was expressed as the amount of enzyme that releases 1 μmol of galacturonic acid in 1 min under the mentioned conditions.

### Effect of incubation period, temperature, pH, inoculum volume, and pectin concentration in pectinase production

The bacterium was cultured in YEP, pectinase production media, for different incubation periods (24–120 h). At different incubation periods, the cultured broth was aseptically taken in a sterile Eppendorf tube and centrifuged. The cell-free supernatant was used for enzyme activity assay. The fermentation condition for the bacteria regarding incubation temperatures and pH was studied by culturing at different incubation temperatures (30°C, 35°C, 40°C, and 45°C) and pH (5, 6, 7, 8, 9, and 10). Similarly, the different inoculum volume (0.5, 1, 2, 3, and 4% v/v), and different pectin concentrations (0.5, 1, 1.5, and 2% w/v) were added to the media, to study the enzyme activity for 96 h.

### Agro-waste preparation and multi-enzyme production

Different agro-wastes which are easily and locally available were selected. Orange peel, banana peel, pomegranate peel, and pumpkin pulp+seeds were from the waste of those fruits and vegetables bought from the market. Barley straw and maple leaf were commercially available. Canola straw was accumulated from a local farm, and brewer’s spent grains from a local brewing company (Sleeping Giant Brewing Co., Thunder Bay, Ontario, Canada). All those agro-wastes were dried, ground in a coffee grinder, and washed with hot water several times to remove contaminant and simple sugar. The presence of reducing sugar was determined by DNS method. Once the samples were free of reducing sugar, they were dried on a hot air oven at 50°C for 48 h (till constant weight). The dried agro-waste powders were kept in airtight containers for further use.

For multi-enzymes production using agro-wastes, the bacterium was cultured on the media containing 1% w/v agro-wastes as the carbon source instead of 1% w/v pectin in the YEP pectinase production media. Polysaccharides such as pectinase, polygalacturonase (PGase), xylanase, and cellulase activities were determined every 24 h. The citrus pectin, polygalacturonic acid, beechwood xylan, and carboxymethyl cellulose (CMC) were used as the respective substrates.

#### Optimization of cultural conditions for the maximum enzyme production

Orange peel was illustrated as the best substrate among the agro-wastes used in this study, so optimization of enzyme production was performed by using orange peel. Plackett–Burman design included seven different factors at two levels; incubation temperature, pH, incubation period, MgSO_4_, NaCl, FeSO_4_, and (NH_4_)_2_SO_4_ to screen the main influencing factor, including 15 experimental runs with three central points. The high level (+1) indicates the maximum concentration, and the low level (−1) indicates the minimum concentration of the variables (Table 1). The Box–Behnken design (BBD) response surface methodology was used to optimize the cultural conditions for maximum enzyme production using the most significant factors from the Plackett–Burman design. Here, enzyme activity was considered response variable, whereas incubation period (h), orange peel concentration (% w/v), and inoculum volume (% v/v) were three independent variables. The BBD used all the factors at three levels assigned as −1, 0, and +1 for the lowest, central, and highest value (Table 2). Both Plackett–Burman and BBD experimental designs were generated by using the Minitab 16 software.

**Table 1:** Experimental design and enzymes activities (U/mL) using Plackett–Burman factorial design.

Run	Temperature (°C)	pH	Time (h)	MgSO_4_ (%w/v)	NaCl (%w/v)	Fe_2_SO_4_ (%w/v)	(NH_4_)_2_SO_4_ (%w/v)	Pectinase (U/ml)	PGase (U/ml)	Xylanase (U/ml)	Cellulase (U/ml)
1	40	6	96	0.02	0.1	0.01	0.5	6.54	4.98	3.39	3.10
2	40	9	12	0.05	0.1	0.01	0.1	2.25	3.72	1.46	0.00
3	30	9	96	0.02	0.5	0.01	0.1	5.01	5.25	5.84	0.00
4	40	6	96	0.05	0.1	0.05	0.1	5.91	8.01	6.26	7.26
5	40	9	12	0.05	0.5	0.01	0.5	1.99	3.60	0.71	0.00
6	40	9	96	0.02	0.5	0.05	0.1	3.73	1.85	0.00	0.00
7	30	9	96	0.05	0.1	0.05	0.5	6.60	5.60	8.25	6.96
8	30	6	96	0.05	0.5	0.01	0.5	5.69	5.49	7.25	4.15
9	30	6	12	0.05	0.5	0.05	0.1	2.39	3.00	1.83	0.00
10	40	6	12	0.02	0.5	0.05	0.5	2.03	2.75	1.19	0.00
11	30	9	12	0.02	0.1	0.05	0.5	2.34	2.50	2.60	0.00
12	30	6	12	0.02	0.1	0.01	0.1	1.51	1.89	0.71	0.00
13	35	7.5	54	0.035	0.3	0.03	0.3	7.42	7.13	8.13	6.00
14	35	7.5	54	0.035	0.3	0.03	0.3	6.15	6.78	5.77	5.47
15	35	7.5	54	0.035	0.3	0.03	0.3	5.92	5.71	5.52	6.88

**Table 2:** Box–Behnken Design for enzymes production by using orange peel as the substrate [−1, 0, and +1 are the codes for the variables].

Run	Time (h)	Orange peel (% w/v)	Inoculum volume (% v/v)	Pectinase (U/ml)		PGase (U/ml)	Xylanase (U/ml)		Cellulase (U/ml)
				Observed	Predicted	Observed	Observed	Predicted	Observed
1	12 (−1)	0.5 (−1)	2 (0)	1.71	2.60	1.25	0.96	1.27	0.00
2	36 (+1)	0.5 (−1)	2 (0)	7.41	8.33	5.65	8.59	8.41	0.00
3	12 (−1)	1.5 (+1)	2 (0)	4.58	3.66	7.98	1.03	1.21	13.68
4	36 ( + 1)	1.5 (+1)	2 (0)	6.53	5.64	6.12	7.09	6.77	17.22
5	12 (−1)	1 (0)	1 (−1)	2.34	2.21	0.16	2.67	2.52	0.00
6	36 (+1)	1 (0)	1 (−1)	5.52	5.37	5.87	8.68	9.02	8.8
7	12 (−1)	1 (0)	3 (+1)	1.01	1.16	1.48	0.00	0.00	0.00
8	36 (+1)	1 (0)	3 (+1)	5.57	5.70	7.27	5.72	5.87	13.69
9	24 (0)	0.5 (−1)	1 (−1)	5.24	4.47	8.24	6.57	6.41	7.04
10	24 (0)	1.5 (+1)	1 (−1)	4.49	5.54	4.62	8.23	8.20	17.34
11	24 (0)	0.5 (−1)	3 (+1)	7.04	5.99	7.43	6.03	6.05	7.51
12	24 (0)	1.5 (+1)	3 (+1)	2.54	3.30	0.11	2.38	2.54	0.00
13	24 (0)	1 (0)	2 (0)	10.62	9.49	13.83	16.79	16.27	19.54
14	24 (0)	1 (0)	2 (0)	8.15	9.49	10.66	15.75	16.27	17.90
15	24 (0)	1 (0)	2 (0)	9.69	9.49	11.25	16.27	16.27	18.72

### Determination of lipid content

The bacterium was cultured in yeast extract peptone (YEP’) culture media (consisting of 0.25, 0.25, 0.15, 2% w/v of yeast extract, peptone, MgSO_4_, dextrose, respectively) and Mineral salt medium (MSM) culture media (consisting of 0.9, 0.15, 0.02, 0.01, 0.00012, 0.002, and 0.005% w/v of Na_2_HPO_4_, KH_2_PO_4_, MgSO_4_, (NH_4_)_2_SO_4_, ferric citrate, CaCl_2_, and NaHCO_3_, respectively). Every 24 h, lipid content was determined by Bligh and Dyer (1959) with little modification. In brief, the biomass was first harvested by centrifugation at 10,000 g for 10 min, and 1 gm of the pellet was sonicated and then homogenized with a 20 ml mixture containing chloroform, methanol, and water (1:2:0.8 ratio), followed by 20 min of shaking in an orbital shaker at ambient room temperature. The homogenate was centrifuged at low speed (2,000 g) to separate two phases. The upper phase was siphoned off, and the lower chloroform phase containing lipids was evaporated. Finally, the lipids extracted were quantified by weighing, and lipid content (%) was calculated as,


$$\mathrm{Lipid content}\;\left(\%\right)=\frac{\mathrm{Weight of lipid}\;\mathrm{per}\;\mathrm{mL}}{\mathrm{Dry}\;\mathrm{weight of bacteria}\;\mathrm{per}\;\mathrm{mL}}\times 100$$

Furthermore, lipid content was determined at two different media (YEP’ and MSM), different incubation periods (24 to 96 h), temperature (30°C–45°C) and pH values (6–9) to optimize the lipid content from the bacterium.

### Statistical analysis

All the tests were performed in triplicates, and the data were expressed as mean with standard deviation (mean ± SD). One-way analysis of variance (ANOVA) was used to assess the statistical significance, followed by the Tukey–Kramer comparison. The data is regarded as statistically significant when the *p*-value of the experimental data is less than 0.05.

## Results

### Isolation and identification of the bacterium

After subculturing many times, pure and isolated colony was observed. The colony morphology of an isolated colony on the Sabouraud dextrose agar (SDA), Casein starch agar (CSA), and Nutrient agar (NA) plates were observed (Supplementary Figure 1). The colony of the bacterium was pale in color, irregular in shape, has many colorless waterlike droplets on SDA agar. The peripheral of the colony was soft to pick, whereas the inner part was hard. The unique colony characteristics with the occurrence of waterlike droplets suspected the bacterium of producing lipids.

**Figure 1:**
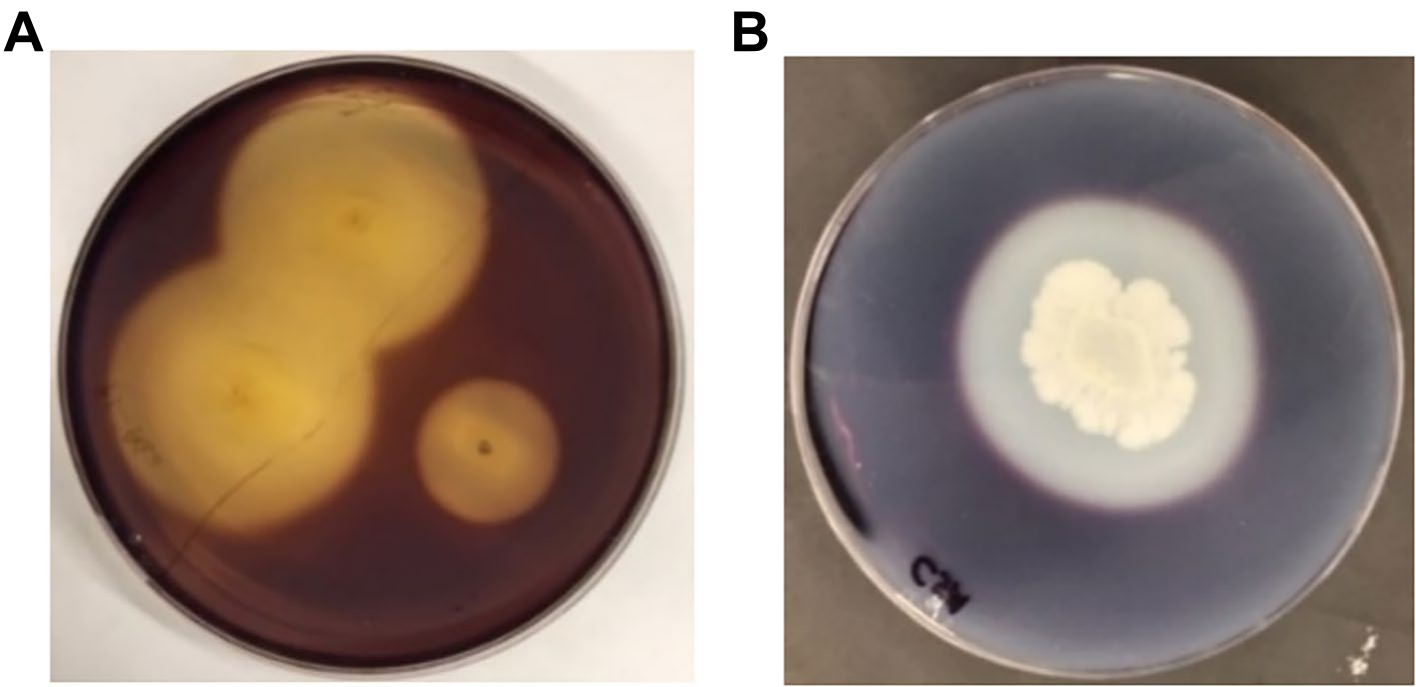
Screening test for **(A)** pectinase, and **(B)** amylase.

Then the bacterium was screened for pectinase, cellulase, xylanase, lipase, and amylase enzymes. The clear zone around the colony was observed, indicating the bacterium was strongly positive for pectinase and amylase screening tests (Figures 1A,B), whereas weakly positive for cellulase, xylanase and lipase.

**Figure 2:**
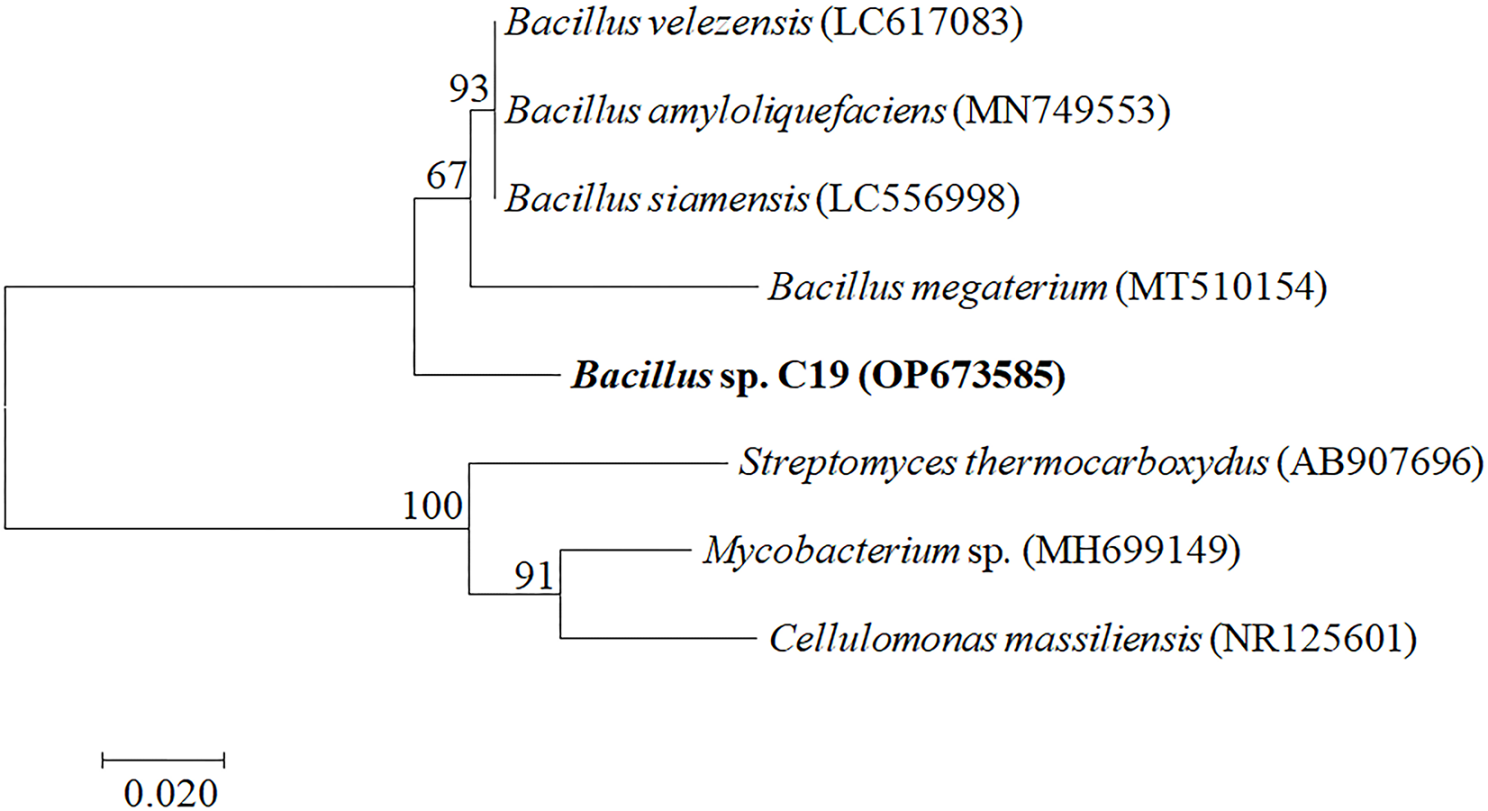
Phylogenetic relationships of the bacterium constructed by Neighbor-Joining (NJ) tree based on 16S rDNA gene sequences. Numbers present on branches of the NJ tree are bootstrap support values.

The bacterium was identified as *Bacillus* sp. (accession number OP673585) after isolation of the bacterium, DNA extraction, gel electrophoresis, 16S rDNA sequencing, and phylogenetic analysis. The 16S rDNA amplification and detection were performed as mentioned in the method, and the result of the 16S rDNA after gel electrophoresis depicted the fragments at around 1,500 bp (Supplementary Figure 2). The sequence similarity of the isolate was compared with the known species and similar sequences in the NCBI database. The phylogenetic tree of 16S rDNA sequences based on the Neighbor-Joining algorithm was constructed, as shown in Figure 2. The constructed phylogenetic tree depicted the studied bacterium (*Bacillus* sp. C19) is closely related to the other *Bacillus* sp. (*Bacillus megaterium, Bacillus siamensis, Bacillus amyloliquefaciens,* and *Bacillus velezensis*; Figure 2).

The uncorrected genetic divergence between the bacterium and topotypic *Bacillus siamensis, Bacillus velezensis, and Bacillus amyloliquefaciens* was 3.5%, whereas *Mycobacterium* sp., *Cellulomonas massiliensis*, and *Streptomyces thermocboxydus* were 20, 21, and 21%, respectively, (shown in Supplementary Table 1).

### Effect of incubation period, temperature, inoculum volume, pH, and pectin concentration in pectinase activity

**Figure 3:**
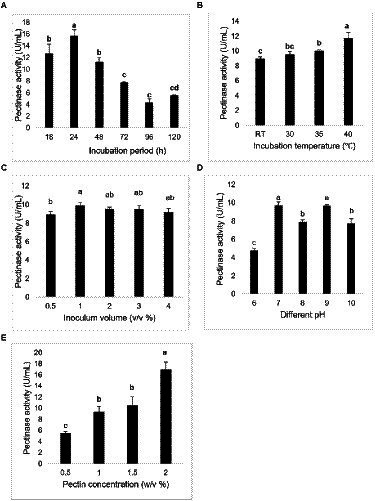
Pectinase activity at different **(A)** incubation period (h), **(B)** temperature (°C), **(C)** inoculum volume (% v/v), **(D)** pH, and **(E)** pectin concentration (% w/v). The bar represented the mean pectinase activity with the standard deviation error bar at different conditions. Lowercase alphabets indicate that the values are statistically significant at *p* ≤ 0.05.

The maximum pectinase activity was observed in incubation period of 24 h, 40°C, 1% v/v inoculum volume, alkaline pH (7 and 9), and pectin 2% w/v (Figure 3). The values observed were statistically significant (*p* ≤ 0.05) when analyzed with one-way analysis of variance (ANOVA) followed by Tukey’s comparisons.

### Potential of *Bacillus* sp. to produce multi-enzymes using agro-wastes

**Figure 4:**
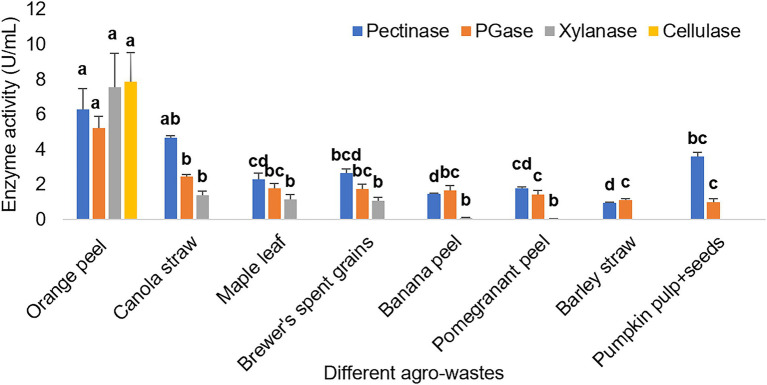
Different enzyme activities exhibited by *Bacillus* sp. using different agro-wastes as carbon sources. The bar represented the mean enzyme activity with the standard deviation error bar. Lowercase alphabets indicate that the values are statistically significant at *p* ≤ 0.05.

Figure 4 depicts the pectinase activity by *Bacillus* sp. using eight agro-wastes. The bacterium could not show xylanase activity from barley straw and pumpkin pulp + seeds, while cellulase activity was only observed from orange peel.

#### Optimization of the cultural conditions for maximum enzyme activities using orange peel

After the observations of different agro-wastes utilization to produce the enzymes, the orange peel was found to be the best substrate for multi-enzymes production. Therefore, the orange peel was selected to optimize the cultural conditions.

##### Plackett–Burman design

Plackett–Burman design was used for the initial statistical screening of seven cultural components (variables) and the enzyme activities (response), and the results are depicted in Table 1. The detailed analysis of the individual factors revealed that the incubation period as an influencing factor for pectinase, PGase, xylanase and cellulase activity with the lowest *p*-value. At the same time, all the six factors, like incubation period, pH, NaCl, Fe_2_SO_4_, MgSO_4_, and (NH_4_)_2_SO_4_ played important roles in cellulase activity. However, they were not significantly influential for other enzyme activities (Supplementary Table 2; Supplementary Figure 4).

##### Box–Behnken design

The optimization of cultural conditions was again designed using Box–Behnken design (BBD) and analyzed in Minitab 16 software. Enzyme activities exhibited by the bacterium using BBD illustrated in Table 2.

The accuracy and statistical significance of each term in the model were evaluated using Analysis of variance (ANOVA). The BBD experimental results of the current study were fitted with a second-order polynomial equation using a multiple regression technique and expressed as:

Pectinase activity (U/mL) *= 9.49 + (1.92 • X1) + (−0.41 • X2) + (−0.18 • X3)* + *(−0.94 • X1 • X2) + (0.35 • X1 • X3) + (−0.94 • X2 • X3)* + *(−2.82 • X1^2^)* + *(−1.60 • X2^2^)* + *(−3.05 • X3^2^).*
PGase activity (U/mL) = 11.91 + (1.75 • X1) + (−0.47 • X2) + (−0.32 • X3) + (−1.56 • X1 • X2) + (0.02 • X1 • X3) + (−0.92 • X2 • X3) + (−4.03 • X1^2^) + (−2.63• X2^2^) + (−4.18 • X3^2^).
Xylanase activity (U/mL) = 16.27 + (3.31 • X1) + (−0.43 • X2) + (−1.64 • X3) + (−0.39 • X1 • X2) + (0.19 • X1 • X3) + (−1.33 • X2 • X3) + (−6.82 • X1^2^) + (−5.03• X2^2^) + (−5.44 • X3^2^).
Cellulase activity (U/mL) = 18.72 + (4.51 • X1) + (4.33 • X2) + (−1.83 • X3) + (0.57• X1 • X2) + (0.98 • X1 • X3) + (−5.36 • X2 • X3) + (−7.89 • X1^2^) + (−4.25• X2^2^) + (−7.41 • X3^2^).

X1 represents time, X2 orange peel concentration, and X3 inoculum volume.

The *p*-values for pectinase, PGase, xylanase and cellulase activity were 0.03, 0.35, 0.000, 0.35 and lack of fit 0.434, 0.128, 0.892, and 0.01, respectively. The R^2^ values for pectinase, PGase, xylanase and cellulase were observed as 0.91, 0.73, 0.99, and 0.72, respectively. The regression p-value and lack of fit observed from the analysis of variance revealed that the quadratic equations were relatively reliable for evaluating bacterium’s pectinase and xylanase activities (Table 3).

**Table 3:** Analysis of Variance (ANOVA) for BBD quadratic model.

Response	Terms of model	Degree of freedom	Sum of square	Mean square	*F*-Value	*P*-Value
Pectinase	Constant	9	103.39	11.48	5.84	0.03^*^
PGase			171.79	19.09	1.47	0.35
Xylanase			444.04	49.34	350.86	0.00^*^
Cellulase			898.93	99.88	1.46	0.35
Pectinase	Incubation period (X1)	1	29.64	29.64	15.29	0.05^*^
PGase			24.62	24.62	1.9	0.23
Xylanase			87.61	49.34	623.01	0.00^*^
Cellulase			162.67	99.88	2.38	0.18
Pectinase	Orange peel % (X2)	1	1.33	1.33	0.68	0.45
PGase			1.76	1.76	0.14	0.73
Xylanase			1.46	1.46	10.36	0.02^*^
Cellulase			150.04	150.04	2.2	0.19
Pectinase	Inoculum volume (X3)	1	0.25	0.25	0.13	0.73
Pgase			0.85	0.85	0.07	0.81
Xylanase			21.39	21.39	152.1	0.00^*^
Cellulase			26.69	26.69	0.39	0.56
Pectinase	X1^2^	1	23.15	29.43	14.97	0.01^*^
PGase			46.98	60.08	4.63	0.08
Xylanase			137.77	171.89	122.12	0.00^*^
Cellulase			186.27	23.24	3.37	0.37
Pectinase	X2^2^	1	6.96	9.5	4.83	0.08
PGase			19.76	25.56	1.96	0.22
Xylanase			78.87	93.28	663.39	0.00^*^
Cellulase			50.35	66.75	0.98	0.13
Pectinase	X3^2^	1	34.46	34.46	17.53	0.01^*^
PGase			64.64	64.64	4.98	0.08
Xylanase			109.15	109.15	776.25	0.00^*^
Cellulase			202.71	202.71	2.97	0.15
Pectinase	X1.X2	1	3.51	3.51	1.79	0.64
PGase			9.78	9.78	0.75	0.99
Xylanase			0.62	0.62	43.8	0.36
Cellulase			1.31	1.31	0.02	0.82
Pectinase	X2.X3	1	0.48	0.48	0.24	0.64
PGase			0	0	0	0.99
Xylanase			0.15	0.15	1.03	0.36
Cellulase			3.89	3.89	0.06	0.82
Pectinase	X1.X3	1	3.53	3.53	1.8	0.24
PGase			3.4	3.4	0.26	0.63
Xylanase			7.03	7.03	49.97	0.00^*^
Cellulase			114.99	114.99	1.68	0.25
Pectinase	Lack of fit	3	6.73	2.24	1.45	0.43
PGase			59.24	19.75	6.96	0.13
Xylanase			0.16	0.05	0.2	0.89
Cellulase			340.18	113.39	168.51	0.01^*^
Pectinase	Pure error	2	3.1	1.55		
PGase			5.68	2.84		
Xylanase			0.54	0.27		
Cellulase			1.35	0.67		
Pectinase	R^2^	0.91				
PGase		0.73				
Xylanase		0.99				
Cellulase		0.72				

* Significant values at *p* ≤ 0.05.

**Figure 5:**
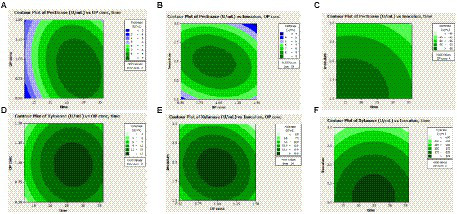
Contour plot showing the interaction effects of orange peel concentration (OP conc), Incubation period (hour), and inoculum volume (Inoculum) on pectinase **(A–C)** and xylanase **(D–F)** activities.

The Contour plot Figures 5A,D showed the interaction of orange peel concentration and incubation period, (Figures 5B,E) the interaction of inoculum volume and orange peel concentration, and (Figures 5C,F) showed the interaction of inoculum volume and incubation period for pectinase and xylanase activities, respectively.

Figure 5 depicted that both pectinase and xylanase activities increased with an increase in orange peel concentration and incubation period to certain values and decreased in activities after the optimal values. Figures 5C,F illustrated a low inoculum volume and moderate incubation period gave the maximum pectinase and xylanase activities.

From the response optimizer, the optimal conditions observed for pectinase activity were incubation period of 29 h, 1% w/v orange peel concentration, and 2% v/v inoculum volume. In contrast, the optimal conditions for xylanase activity were 27 h, 1% w/v orange peel concentration, and 2% v/v inoculum volume (Supplementary Figure 5).

The model correctness was determined by performing the experiments under optimal conditions (from response optimizer), and the pectinase and xylanase activities observed were 8.62 ± 1.56 U/ml and 15.02 ± 1.23 U/ml, respectively. However, from BBD, 9.49 U/ml and 16.27 U/ml were revealed as the maximum pectinase and xylanase activities, respectively. At the same time, 9.90 U/ml and 16.76 U/ml were suggested for pectinase and xylanase by BBD optimizer. The enzyme activities thus observed were almost similar; thus, the models for pectinase and xylanase activities were valid and precise.

### Lipid production by the bacterium at different conditions

**Figure 6:**
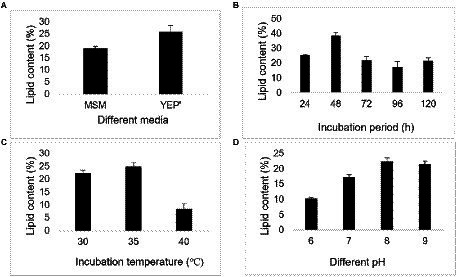
Lipid content exhibited by *Bacillus* sp. at different conditions **(A)** different media, **(B)** different incubation period (h), **(C)** temperature (°C), and **(D)** pH.

The lipid content of the bacterium was around 25% when grown in yeast extract peptone (YEP’) media. Further, the lipid content from the bacterium were studied using parameters like different incubation periods and pH values. The highest lipid contents by the bacterium were observed at incubation period of 48 h, temperature 35°C and pH 8, as illustrated in following figures (Figure 6).

## Discussion

The production of industrially important, novel, and robust enzymes for biotechnological applications is increasing. Therefore, there is a need to isolate and identify the bacteria from natural diversities and optimize the cultural conditions for maximum enzyme production. In our current study, we isolated the bacterium and screened for enzymes. The presence of a relative enzyme hydrolyzes the substrate around the colony, and a clear hydrolysis zone around the colony is observed. However, the substrate not hydrolyzed by relative enzymes forms a complex and does not show a clear zone (Soares et al., 1999; Meddeb-Mouelhi et al., 2014; Shrestha et al., 2021b; Al Mousa et al., 2022). On this basis, the bacterium was pectinase, amylase, cellulase, and xylanase producer.

The contaminant of the broth was identified as bacterium. Only 16S rDNA got amplified and showed the band at 1,500 bp region. Further, the bacterium was identified as *Bacillus* sp. because the phylogenetic tree illustrated the bacterium closely related to the *Bacillus* sp. (*Bacillus megaterium, Bacillus siamensis, Bacillus amyloliquefaciens,* and *Bacillus velezensis*). Furthermore, the uncorrected genetic divergence helped to confirm the bacterium as *Bacillus* sp.

*Bacillus* sp. are the dominant bacteria (Schallmey et al., 2004) and they have irregular colony (De Vos et al., 2015). Our study too revealed the irregular colony of *Bacillus* sp. Additionally, we observed clear water-like droplets on the colony which is unique character. Each bacterium has specific optimal conditions for the maximum enzyme activity. For instance, the study illustrated the optimal conditions by *Bacillus* sp. for the maximum xylanase activity were 50°C, 72 h, pH 7.0 ± 0.2, and 1% xylan concentration (Lawrence et al., 2015). Another study recorded the maximum pectinase activity by *Aspergillus* sp. at 35°C, pH 6.5, 1% citrus pectin and after 192 h of incubation period (Ketipally and Ram, 2018). Similarly*, Streptomyces thermocoprophilus* demonstrated maximum cellulase and xylanase activity at 40°C, pH 6.5, 120 h, and 1% alkaline peroxide pretreated empty fruit bunch (Sinjaroonsak et al., 2020). And our study observed the highest pectinase activity by *Bacillus* sp. in 24 h, 40°C, 1% v/v inoculum volume, alkaline pH (7 and 9), and pectin 2% w/v. The variation in the optimal conditions for enzyme activities may be due to the differences in the microorganisms used. The optimal conditions affect the growth and metabolic rate of the organisms exploited, and the maximum activity is illustrated at the most favorable conditions depending on the organisms.

The cheapest and readily available agro-industrial wastes can be used for industrially important enzyme production (Bharathiraja et al., 2017). The various agro-wastes, when used as the carbon source for enzyme production, the bacterium demonstrated the highest enzyme activities from orange peel. This may be because the bacterium must have found all the favorable conditions from orange peel only to demonstrate all the studied enzyme activities. Also, the orange peel may have a lower lignin concentration, making it inaccessible to pectin, hemicellulose and cellulose for the bacterium (Al Mousa et al., 2022). The bacterium exhibited xylanase and pectinase activity from canola straw, maple leaf, brewer’s spent grains, banana peel, and pomegranate peel. Further, the bacterium showed only pectinase activity from barley straw and pumpkin pulp+seeds. Thus, this study depicts the bacterium’s ability to use orange peel more effectively and efficiently than other agro-wastes and demonstrates orange peel as the best substrate for *Bacillus* sp. Similarly, another study demonstrated that maximum pectinase and cellulase activity by *Mucor circinelloides* and *M. hiemalis* using tangerine peel due to the low concentration of lignin, tissue structure flexibility, and easy access to pectin, cellulose and hemicellulose (Al Mousa et al., 2022). Another study reported that banana peel with high pectin and starch content induced most of the target enzymes by *Aspergillus niger*. In contrast, cellulose-rich sugarcane bagasse induced beta-glucanase and xylanase. At the same time, starch-rich cassava pulp induced amylase and other enzymes but was comparatively lower than banana peel (Laothanachareon et al., 2022). Such variation might be due to the various chemical compositions and carbon sources of agro-wastes which induce the target enzyme production by a microorganism differently.

The different concentrations (0.5, 1, 1.5, and 2% w/v) of agro-wastes were studied for enzyme activities. The enzyme activities were increased with an increase in the concentration of almost all agro-wastes used (orange peel, barley straw, pumpkin pulp + seeds, banana peel, barley spent grains, canola straw, and maple leaf). In contrast, a high concentration of pomegranate peel decreased the pectinase and PGase activities (Supplementary Figures 3A–C). This result relates that the bacterium’s ability to illustrate enzyme activity is directly proportional to agro-waste concentrations except for pomegranate peel. The higher the concentration of agro-wastes, the higher the chance of exposure to relative carbon source (pectin for pectinase, and hemicellulose for xylanase) present in that agro-waste.

Plackett–Burman design is a powerful and unique design to screen, identify and evaluate important variables that affect the response of the experimental tests. Thus, the present study used the Plackett–Burman design as an initial statistical screening of seven cultural components (variables) for enzyme activities (response). Our study revealed incubation period as an influencing factor for pectinase, PGase, xylanase and cellulase activity from this design, with the *p*-value < 0.05. However, the cellulase activity was only affected by all six factors and was not significantly influential for other enzyme activities. Likewise, a study used the Plackett–Burman design and reported the pectinase activity by *Bacillus sonoresis* was strongly affected by the pectin mass fraction, pH, and MgSO_4_ among eight different parameters studied (Mohandas et al., 2018).

Therefore, BBD was again used for optimizing the cultural condition considering incubation period, orange peel concentration and inoculum volume as the independent variables. Generally, the p-value lesser than 0.05 ensures the terms are statistically significant for each coefficient. The relationship between parameters was found significant for pectinase and xylanase but not for PGase and cellulase activities. Further, the statistical significance was checked by F-test to evaluate the coefficient of determination (R^2^). The lesser R^2^ value is not good, so the model was not too good for PGase and cellulase activity. However, the model was perfect for pectinase and xylanase activities.

Due to the depletion of fossil fuel reserves, different alternate sources are being explored (Yusuf, 2019). The microorganisms have potential as an alternative for lipid production as they can accumulate the oil/lipid in them (Patel et al., 2020). The present study explored the capacity of bacterium to produce lipids because it depicted the water like droplets on the SDA media. The study illustrated that the lipid content was higher in YEP’ media, possibly due to the higher cell growth, and the media favored the cell growth. In contrast, MSM inhibited the cell growth resulting in a decrease in lipid content. Our study illustrated that the lipid content continuously increased until 48 h of incubation period and decreased onward, which may be related to cell growth. Similarly, the incubation temperature of 35°C and pH 8 favored the cell growth and resulted in higher lipid content at the respective temperature and pH. However, the bacterial lipid accumulation, lipid composition and even the cell membrane compositions of the same species vary with the environmental conditions of exposure (Sohlenkamp and Geiger, 2015) (Table 4). A study demonstrated that the lipid production was in the range of 25.5%–52.9% of cell dry weight by *Fusarium oxysporum* using the synthetic media containing different sugars (glucose, fructose, and sucrose) alone and mixture of them (Matsakas et al., 2017). Whereas, *Bacillus cereus* accumulated lipid 5%–19% on a dry weight basis using palm oil mill wastewater (Karim et al., 2019). Another study reported that *Lipomyces starkey* could potentially be used as a lipid source and accumulate lipid (12%–29.5%) when cultured in oil mill wastewater-containing media (Yousuf et al., 2010). In addition, a study illustrated the microbial lipid from the yeast *Cryptococcus* sp. using corncob hydrolysate as a raw material (Chang et al., 2015). Therefore, microorganisms can be a potential source of lipid production by bioremediating the wastes. Further, studies relating to the isolation and identification of high lipid-containing microorganisms, the optimization of the culturing condition, compositional analysis, and more are recommended for maximum microbial lipid production.

**Table 4:** Enzyme activity and lipid content exhibited by various organisms.

Organism exploited	Enzyme activity	Lipid content (% dry weight basis)	Conditions	References
*Bacillus* sp.	Pectinase: 9.69 ± 0.15 U/ml	-	40°C, 2% w/v pectin, 1% v/v inoculum volume, pH 9	This study
*Aspergillus oryzae*	Pectinase: 1.6–2.07 IU/ml		35°C, 1% w/v citrus pectin, pH 6	Ketipally and Ram (2018)
*Bacillus* sp.	Xylanase: 7.30–7.85 U/ml	-	50°C, pH 7, 1% w/v xylan	Lawrence et al. (2015)
*Mucor circinelloides*	Pectinase: 38.02 U/ml	-	30°C, pH 7, 9 days, 3 ml of inoculum volume, 5% w/v tangerine peel	Al Mousa et al. (2022)
*Mucor circinelloides*	Cellulase: 37.20 U/ml	-	30°C, pH 7, 5 days, 3 ml of inoculum volume, 5% w/v tangerine peel	Al Mousa et al. (2022)
*M. hiemalis*	Pectinase: 39.72 U/ml	-	30°C, pH 5, 7 days, 3 ml of inoculum volume, 5% w/v tangerine peel	Al Mousa et al. (2022)
*M. hiemalis*	Cellulase: 33.82 U/ml	-	30°C, pH 7, 9 days, 3 ml of inoculum volume, 5% w/v tangerine peel	Al Mousa et al. (2022)
*Bacillus* sp.	Pectinase: 6.28 ± 1.19 U/ml Xylanase: 7.54 ± 1.96 U/ml	-	40°C, 1% w/v orange peel, 1% v/v inoculum volume	This study
*Bacillus* sp.	Pectinase: 9.49 ± 1.24 U/ml Xylanase: 16.27 ± 0.72 U/ml	-	BBD (24 h, 1% w/v orange peel, 2% v/v inoculum volume, 40°C)	This study
*Bacillus* sp.	-	22.51 ± 1.12	48 h, 35°C, pH 8, and YEP’ media	This study
*Fusarium oxysporum*	-	25.5–52.9	Containing different sugars (glucose, fructose, sucrose) alone and mixture of them	Matsakas et al. (2017)
*Fusarium oxysporum*	-	17.7–22.0	Containing different concentrations of sweet sorghum	Matsakas et al. (2017)
*B. cereus*	-	5–19	Containing palm oil mill wastewater	Karim et al. (2019)
*Cryptococcus* sp.	-	30	When cultured in yeast malt agar	Chang et al. (2015)

## Conclusion

The pectinase-producing bacterium isolated from the contaminated broth was identified as *Bacillus* sp. from 16S rDNA sequence analysis. The bacterium produced different polysaccharides degrading enzymes, such as pectinase, polygalacturonase, xylanase, and cellulase. However, the different enzyme activities vary with agro-wastes used as low-cost substrates. The response surface methodology illustrated *Bacillus* sp. to exhibit maximum pectinase at 40°C, 29 h of incubation period, 1% w/v orange peel concentration, and 2% v/v inoculum volume. In contrast, the optimal conditions for xylanase activity were 40°C, 27 h, 1% w/v orange peel concentration, and 2% v/v inoculum volume. Furthermore, the bacterium has the potential to produce bacterial lipids. Therefore, the bacterium is a good candidate for producing biotechnologically important multi-enzymes and agro-waste degradation. Since the bacterium illustrated lipid content, it can be a potential feedstock in producing renewable biofuels and environmental resilience.

## Data availability statement

The original contributions presented in the study are included in the article/Supplementary material, further inquiries can be directed to the corresponding author.

## Author contributions

SS designed the experiments, performed majority of experiments, analyzed the data, and drafted the manuscript. JK, CC, and AK helped and coordinated in the entire study. WQ provided the resources, supervised, and did major review of the manuscript. All authors contributed to the article and approved the submitted version.

## Funding

This project was supported by the Natural Science and Engineering Research Council of Canada (NSERC; RGPIN-2017-05366) to WQ and Ontario Graduate Scholarship (OGS) to SS.

## Conflict of interest

The authors declare that the research was conducted in the absence of any commercial or financial relationships that could be construed as a potential conflict of interest.

## Publisher’s note

All claims expressed in this article are solely those of the authors and do not necessarily represent those of their affiliated organizations, or those of the publisher, the editors and the reviewers. Any product that may be evaluated in this article, or claim that may be made by its manufacturer, is not guaranteed or endorsed by the publisher.

## Footnotes

^1^ https://datatopics.worldbank.org/what-a-waste/trends_in_solid_waste_management.html
