# Supplementary material for: Optimization of multiple enzymes production by fermentation using lipid-producing Bacillus sp
Source: Front Microbiol. 2022 Nov 1;13:1049692. doi: 10.3389/fmicb.2022.1049692 (PMC9663924; doi:10.3389/fmicb.2022.1049692)
Supplement: Supplementary file 1 [file Data_Sheet_1.docx]

Supplementary Material

# Supplementary Figures and Tables

## Supplementary Figures

**(A)**
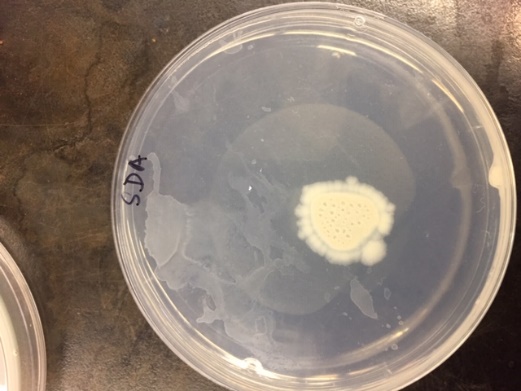
 **(B)**
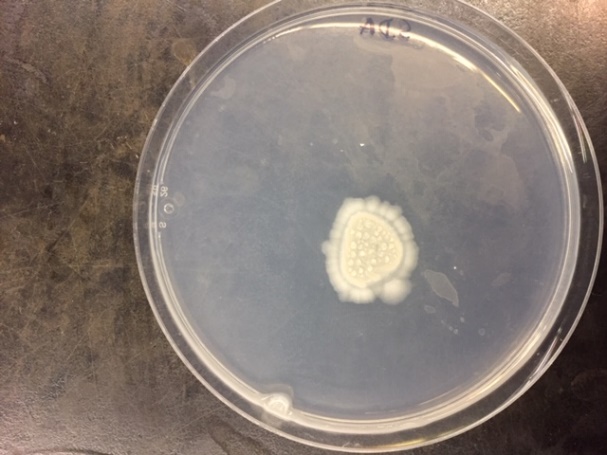


**(C)**
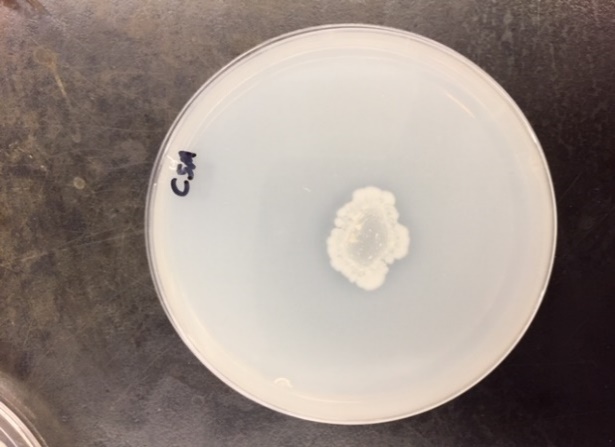
 **(D)**
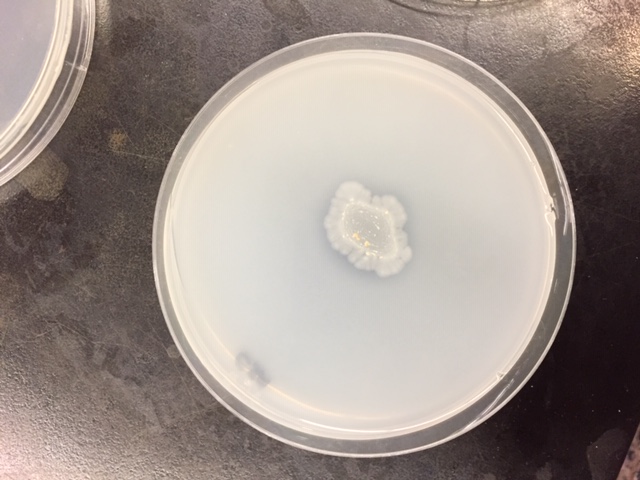


**(E)**
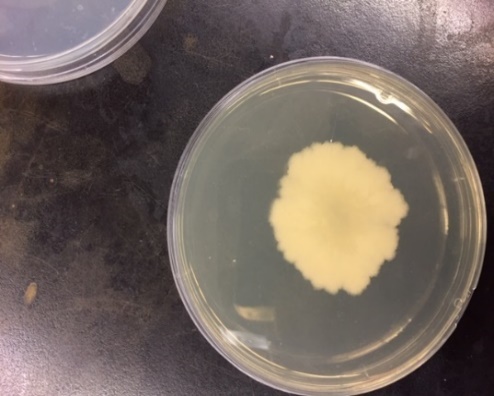
 **(F)**
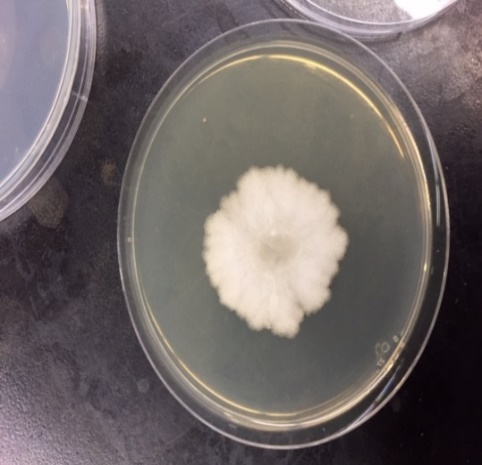


**Supplementary Figure 1** Colony morphology from the back (**A**, **C**, and **E**) and top (**B**, **D**, and **F**) of the plate on Sabouraud Dextrose Agar (SDA), Casein starch agar (CSA), and Nutrient agar (NA), respectively

**
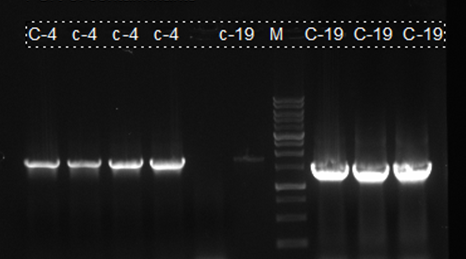
**

**Supplementary Figure 2** 16S rDNA on Gel electrophoresis

**Supplementary Figure 3A** Pectinase activity at different biomass concentrations exhibited by *Bacillus* sp.

**Supplementary Figure 3B** PGase activity exhibited by different biomass concentrations exhibited by *Bacillus* sp.

**Supplementary Figure 3C** Xylanase activity at different biomass concentrations exhibited by *Bacillus* sp.

**(A)**
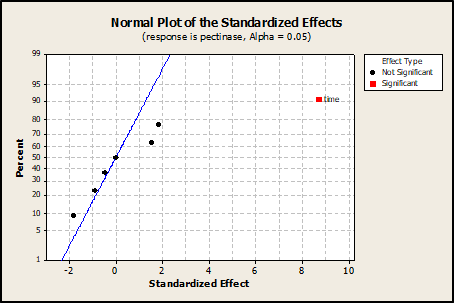
 **(B)**
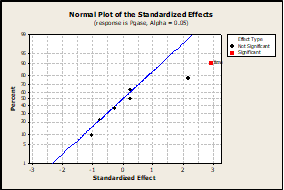


**(C)**
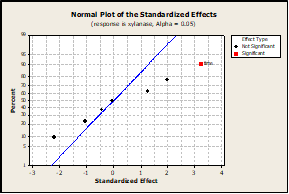
 **(D)**
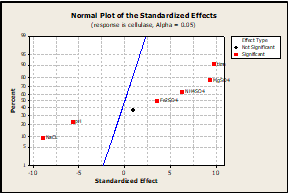


**Supplementary Figure 4** Normal plot of different factors in Plackett-Burman design for **(A)** pectinase, **(B)** PGase, **(C)** xylanase, and **(D)** cellulase

**(A)** **(B)**

**Supplementary Figure 5** The optimal conditions from the response optimizer for **A)** Pectinase and **B)** Xylanase activities

## Supplementary Tables

**Supplementary Table 1** Uncorrected genetic distance between the species

| **S.N** | **Name of species** | **1** | **2** | **3** | **4** | **5** | **6** | **7** | **8** |
| --- | --- | --- | --- | --- | --- | --- | --- | --- | --- |
| 1 | *Bacillus_*sp*.* | 0.000 |  |  |  |  |  |  |  |
| 2 | *Bacillus siamensis_(LC556998)* | 0.035 |  |  |  |  |  |  |  |
| 3 | *Bacillus velezensis_(LC617083)* | 0.035 | 0.000 |  |  |  |  |  |  |
| 4 | *Bacillus amyloliquefaciens (MN749553)* | 0.035 | 0.000 | 0.000 |  |  |  |  |  |
| 5 | *Mycobacterium* sp._(*MH699149)* | 0.206 | 0.198 | 0.198 | 0.198 |  |  |  |  |
| 6 | *Bacillus megaterium_(MT510154)* | 0.088 | 0.051 | 0.051 | 0.051 | 0.223 |  |  |  |
| 7 | *Cellulomonas massiliensis_(NR125601)* | 0.214 | 0.206 | 0.206 | 0.206 | 0.054 | 0.247 |  |  |
| 8 | *Streptomyces thermocarboxydus (AB907696)* | 0.211 | 0.202 | 0.202 | 0.202 | 0.081 | 0.235 | 0.088 | 0.000 |

**Supplementary Table 2** Statistical analysis of Plackett-Burman design showing coefficient values, t- and p-values for each variable on enzymes activities [Notes: ^A^ Pectinase activity, ^B^ PGase activity, ^C^ Xylanase activity, ^D^ Cellulase activity; *Significant values at p≤0.05]

| **Variable** | | **Effect** | **Coefficient** | **t-value** | **p-value** |
| --- | --- | --- | --- | --- | --- |
| **Constant** | A  B  C  D |  | 3.83  4.05  3.19  -0.73 | 19.14  10.42  5.78  -2.98 | 0.00*  0.00*  0.00*  0.02* |
| **Temperature** | A  B  C  D | -0.18  0.19  -2.43  0.45 | -0.09  0.09  -1.22  0.22 | -0.45  0.25  -2.20  0.92 | 0.67  0.81  0.07  0.39 |
| **pH** | A  B  C  D | -0.35  -0.59  -0.49  -2.79 | -0.18  -0.29  -0.24  -1.39 | -0.89  0.25  -0.44  -5.72 | 0.41  0.47  0.67  0.00* |
| **Time** | A  B  C  D | 3.49  2.28  3.56  4.79 | 1.75  1.14  1.78  2.39 | 8.73  2.94  3.22  9.79 | 0.00*  0.03*  0.02*  0.00* |
| **MgSO_4_** | A  B  C  D | 0.61  1.70  2.19  4.58 | 0.31  0.85  1.09  2.29 | 1.53  2.19  1.98  9.36 | 0.18  0.07  0.09  0.00* |
| **NaCl** | A  B  C  D | -0.72  -0.79  -1.16  -4.36 | -0.36  -0.39  -0.58  -2.18 | -1.80  -1.02  -1.05  -8.92 | 0.12  0.34  0.33  0.00* |
| **Fe_2_SO_4_** | A  B  C  D | 0.00  -0.20  -0.06  1.72 | 0.00  -0.10  -0.03  0.86 | 0.00  -0.26  -0.05  3.25 | 0.99  0.80  0.96  0.01* |
| **(NH_4_)_2_SO_4_** | A  B  C  D | 0.73  0.19  1.41  3.08 | 0.37  0.09  0.70  1.54 | 1.83  0.26  1.27  6.30 | 0.12  0.81  0.25  0.00* |
| **Lack of fit** | A  B  C  D | 0.69  0.19  0.34  0.42 | | | |

## Media compositions

| Pectinase screening agar | | |  | Nutrient agar | | |
| --- | --- | --- | --- | --- | --- | --- |
| Components | | Percentage |  | Components | | Percentage |
| Ammonium sulphate | | 0.2 |  | Peptone | | 0.5 |
| Yeast extract | | 0.1 |  | Beef extract/ yeast extract | | 0.3 |
| Na_2_HPO_4_ | | 0.6 |  | NaCl | | 0.5 |
| KH_2_PO_4_ | | 0.3 |  | Agar | | 1.5 |
| Citrus pectin | | 0.5 |  |  | |  |
| Agar | | 2 |  |  | |  |
|  | |  |  |  | |  |
| Xylanase screening agar | | |  | Amylase screening agar | | |
| Components | Percentage | |  | Components | Percentage | |
| Xylan | 0.5 | |  | Starch | 1 | |
| Yeast extract | 0.5 | |  | (NH4)_2_SO_4_ | 0.2 | |
| Peptone | 0.5 | |  | Peptone | 0.5 | |
| MgSO_4_.7H_2_O | 0.02 | |  | MgCl_2_ | 0.01 | |
| K_2_HPO_4_ | 0.1 | |  | K_2_HPO_4_ | 0.2 | |
| Agar | 2 | |  | KH_2_PO_4_ | 0.1 | |
|  |  | |  | Agar | 2 | |
|  |  | |  |  |  | |
| Cellulase screening agar | | |  | Lipase screening agar | | |
| Components | | Percentage |  | Components | | Percentage |
| CMC | | 0.5 |  | NaCl | | 0.5 |
| Yeast extract | | 0.5 |  | CaCl_2_ | | 0.1 |
| Peptone | | 0.5 |  | Peptone | | 1.5 |
| MgSO_4_.7H_2_O | | 0.02 |  | Tween 80 | | 1 |
| K_2_HPO_4_ | | 0.1 |  | Agar | | 2 |
| Agar | | 2 |  | Tween sterilized separately and added later aseptically (pH )6.8 | | |
|  | |  |  |  | | |
| Pectinase production media | | |  | Luria-Bertani (LB) broth | | |
| Components | Percentage | |  | Components | Percentage | |
| Citrus Pectin | 1 | |  | Yeast extract | 1 | |
| Yeast extract | 0.3 | |  | Peptone | 1 | |
| K_2_NO_3_ | 0.2 | |  | NaCl | 0.5 | |
| KH_2_PO_4_ | 0.2 | |  |  |  | |
| K_2_HPO_4_ | 0.2 | |  |  |  | |
